# Nanotechnology as a Tool for Optimizing Topical Photoprotective Formulations Containing Buriti Oil (*Mauritia flexuosa*) and Dry *Aloe vera* Extracts: Stability and Cytotoxicity Evaluations

**DOI:** 10.3390/ph16020292

**Published:** 2023-02-14

**Authors:** Maria Cristina Pinheiro Pereira Reis-Mansur, Christian Campos Firmino Gomes, Fiammetta Nigro, Eduardo Ricci-Júnior, Zaida Maria Faria de Freitas, Elisabete Pereira dos Santos

**Affiliations:** LADEG Laboratório de Desenvolvimento Galênico, Faculty of Pharmacy, Universidade Federal do Rio de Janeiro (UFRJ), Avenida Carlos Chagas Filho, 373, Centro de Ciências da Saúde (CCS), Cidade Universitária, Ilha do Fundão, Rio de Janeiro 21941-902, Brazil

**Keywords:** nanoemulsions, photoprotection, *Aloe vera*, buriti oil, cytotoxicity, stability

## Abstract

Human beings are actively exposed to ultraviolet (UV) radiation, which is associated with skin cancer. This has encouraged the continuous search for more effective and safer photoprotective formulations. Along with the application of traditional organic sunscreens, there is a growing interest in “green products” containing natural compounds such as plant extracts and oils. This trend is combined with the use of nanotechnology as a tool for optimizing the vehicles of such compounds. Nanoemulsions (NEs) are suitable for the encapsulation of natural compounds, which improves topical treatment. Therefore, we have developed oil-in-water (O/W) nanoemulsions containing 3% buriti oil (BO), incorporated in a 10% vegetal extract of *Aloe vera* (AV) by means of ultrasonic processing to improve the chemical characteristics of this component and, consequently, its efficacy and safety in pharmaceutical and cosmetic formulations. The composition of the formulation was initially defined in a preliminary study on surfactants where the concentrations of Tween^®^ 80 and Span^®^ 20 were evaluated in relation to particle size and the polydispersity index (PDI). The nanoemulsion was prepared and then chemical sunscreens were incorporated with the aim of developing a sunscreen nanoemulsion called NE-A19. This nanoemulsion was found to be the best formulation due to its stability, droplet size (146.80 ± 2.74), and PDI (0.302 ± 0.088), with a monomodal size distribution. The stability was evaluated over 90 days and showed a low growth in particle size at the end of the study. NE-A19 exhibited good viscosity and organoleptic properties, in addition to an occlusion factor indicating an interesting and higher water holding capacity when compared with a NE without AV (*p* < 0.05). The in vitro efficacy and safety studies of NE-19A were promising. Its average in vitro sun protection factor value was 49, with a critical wavelength (λ_c_) of 369.7 nm, satisfactory UVA protection, and a UVA/UVB ratio of 0.40, indicating broad spectrum protection against UVA and UVB radiation. Furthermore, NE-19A displayed a good safety profile in dermal keratinocytes. It can be concluded that NE-19A is a promising formulation for carrying natural products, such as buriti oil and AV, associated with synthetic filters in lower concentrations.

## 1. Introduction

Significant advances in nanotechnology have been observed over recent decades, particularly in the pharmaceutical industry, such as the development of nanostructured systems that allow for the encapsulation of active compounds to solve problems of solubility, toxicity, and stability [1,2]. Nanoemulsions consist of oil-in-water (o/w) or water-in-oil (w/o) dispersions, with droplet diameters typically in the range of 10–1000 nm which are non-viscous and kinetically stable Newtonian fluids, depending on the concentrations of the respective phases [3,4]. These nanostructured carrier systems present several advantages, such as higher stability when compared with traditional emulsions, low toxicity, high biocompatibility, and a high potential to incorporate hydrophilic and lipophilic active compounds in the same preparation [5,6]. Additionally, nanoemulsions also present other interesting advantages for industry, such as low viscosity (which facilitates the application of the formulation on the skin), pleasant texture, and nanometric size (which improves skin coverage and occlusive film formation, as well as protects active compounds against oxidative degradation) [7,8].

As a result, nanoemulsions find applications in several industrial segments, including drug and bioactive agent delivery, nutritional supplements, and pesticides [9]. However, these nanostructured systems have some characteristics that deserve attention, such as stability and toxicity [10]. The stability of nanomaterials can be gauged from its physicochemical properties, their interactions and effects on cellular mechanism and uptake, and subcellular confinement [11,12]. The nanotoxicity of a single nanospecies concerns not just the interaction of that species, but also the interaction of its components with different ecosystems and human health [13,14]. To analyze the whole context, in vitro and in vivo stability-related nanotoxicity studies are indispensable.

Solar radiation can induce numerous effects on the human body, some beneficial and harmful, directly or indirectly affecting human health [15,16]. Such realizations have aroused academic interest over the past centuries, and in 1801, Johann Wilhelm Ritter discovered ultraviolet (UV) rays, one of the many types of radiation emitted by the sun that strikes the Earth’s surface. Everard Home investigated the cause of sunburn in 1820, and in 1878 Otto Veiel started to propose photoprotection mechanisms using natural molecules, such as tannins. In 1922, Hausser and Vahle identified the band in the spectrum of light that correlated erythema and sunburn and developed the first sunscreens based on cinnamates and aminobenzoic acid [17,18].

For a better understanding of how these substances exhibit photoprotective action, it is necessary to understand the length range of UV radiation. This radiation composes of different types of rays with wavelengths ranging from 290 to 400 nm, subdivided into UVA (320–400 nm), UVB (280–320 nm), and UVC (100–280 nm) [19]. The latter reaches the Earth in very small amounts because it is almost completely absorbed by stratospheric ozone [20].

Over time, several strategies have been developed to reduce the incidence of UV radiation on the skin and avoid harmful effects, from clothing containing metallic nanoparticles to accessories such as sunshades, but the most common practice is the application of solar filters [21]. These include inorganic filters that act by reflecting UV rays, such as titanium (IV) oxide (TiO_2_) and zinc(II) oxide (ZnO), as well as organic filters, whose mechanism of action is based on the absorption of UV radiation by the aromatic chains of the compounds, such as benzophenones and cinnamates. The absorption by organic filters causes an electronic agitation in the aromatic structure, which subsequently disperses this energy by assuming a more stable configuration [22]. 

The current sunscreen market constantly seeks innovation to stand out from the competition and offer products that better meet consumer needs [23]. This occurs either by developing effective formulations, using few raw materials to reduce costs and facilitate access, or by employing state-of-the-art technology to produce new mechanisms of action and presentation [17]. The development of more effective and safer products for broad spectrum photoprotection, with high sun protection factors (SPFs), requires the development of innovative multifunctional cosmetic formulations through the usage of new technologies and modern delivery systems, capable of conveying the active compounds in the formulations, reducing the risks to the patient, and maximizing its positive effect. Another way is the use of a variety of active ingredients, especially those from plants to produce “green products”, with the possibility of associating these plant-derived ingredients with other substances to produce a synergistic photoprotective effect as well as complementary functions, such as antioxidant, moisturizing, and wetting properties [24,25].

Recently, the main natural active ingredients typically used are dry or glycolic plant extracts and vegetable oils, compositions that concentrate a large number of active substances in small amounts of materials, which are ideal for incorporation in cosmetic formulations, especially semi-solid ones. In view of the vast plant biodiversity in Brasil, plants such as *Aloe vera* (AV) stand out because of their millennia-long presence in traditional medicine in topical applications, such as the use of its mucilage in hair moisturizer. Buriti is also a fruit of cosmetic interest, and buriti oil (BO) is rich in components with antioxidant and photoprotective properties, besides having a very attractive amber coloration [8,26]. 

Therefore, the objective of this work was to develop o/w nanoemulsion systems containing natural active compounds with moisturizing properties, such as dry AV and BO extracts, as well as traditional photoprotective substances, to obtain stable and innovative formulations that are capable of increasing the efficacy against the harmful effects of UV radiation, while ensuring the safety of these active compounds in the skin, by performing stability and cytotoxicity evaluations.

## 2. Results and Discussion

**Figure 1:**
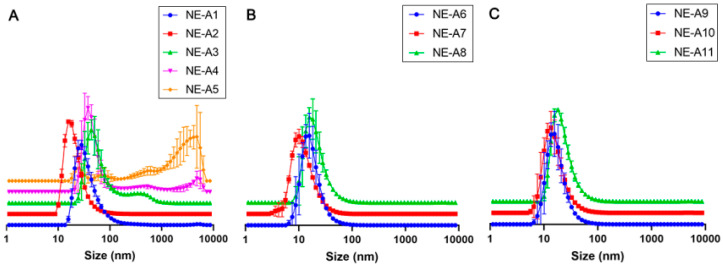
Mean droplet size (nm) distribution curves of the ultrasonically prepared nanoemulsions: (**A**) NE-A1 to NE-A5, (**B**) NE-A6 to NE-A8, and (**C**) NE-A9 to NE-A11.

The prepared NEs show good organoleptic characteristics without phase separation, have a pleasant beige coloration, and a topical use with compatible consistency, as observed with NE-A19 (see on supplementary material Figure S1: NE-A19 nanoemulsion development flowchart). The composition of the NEs shows the usual components and component concentrations, as already described in the literature [8], which also evaluated the BO in photoprotective NEs. Initially, the mean droplet size, mean PDI, and their respective standard deviations were determined for the first eight nanosystems (NE-A1 to NE-A8), developed using ultrasound, with varying surfactant concentration and proportion. Among all evaluated surfactant concentrations, NE-A2, whose composition was 9% *w*/*w* Tween^®^ 80 and 6% *w*/*w* Span^®^ 20, shows the most homogeneous droplet size distribution curve, between NE-A1 and NE-A5 (Figure 1A). On the other hand, NE-A3, NE-A4, and NE-A5 exhibit instability and phase separation profiles within a few minutes of preparation. Therefore, NE-A2 was considered the most promising formulation, but its small average droplet size, relative standard deviation of 47.55 ± 0.25 nm, and PDI of 0.382 posed a potential risk of skin permeation. Consequently, the impact of the total surfactant concentration, in the range of 8–20%, was analyzed, obtaining NE-A6 to NE-A8 in the same proportion as NE-A2. Despite the homogeneity in the distribution curves, the average droplet sizes of NE-A6 to NE-A8 (Figure 1B) are below 50 nm, possibly not safe for topical use as healthy human skin does not allow the absorption of droplets in the order of hundreds of nanometers. Subsequently, an evaluation of the ultrasound equipment parameters was performed to elucidate if the droplet sizes were related to the energy delivered to the system. First, the processing time and its variation was observed for periods of 10 min (NE-A9), 15 min (NE-A10), and 20 min (NE-A11). However, the average droplet sizes are still below the desirable size, according to the size distribution curves (Figure 1C).

Thereafter, the minimum equipment power (20%) was evaluated at even shorter time intervals (0, 1, 2, and 3 min) for NE-A12, NE-A13, NE-A14, and NE-A15, respectively. This procedure demonstrated the sensitivity of the proposed nanostructured systems to energy input, considering that the NE stirred only by vortexing already exhibits a mean droplet size that is in the nanometric range (NE-A12) and the longest use of ultrasound already caused intense droplet breakage (NE-A13 to NE-A15). The employed ultrasonic processing proves to be unable to produce NEs with average droplet sizes between 100 and 300 nm. This droplet size is necessary for promoting the permeation of encapsulated active compounds into the deep skin layers without absorbing these substances. The marked reduction in this parameter could be attributed to the high power of the sound waves and, therefore, the Ultra-Turrax^®^ (Staufen, Germany) was used as a high-energy processing tool. It ruptured the droplets by shearing them with a rotating shaft at high speed. The only process parameter was the number of revolutions per minute. 

**Figure 2:**
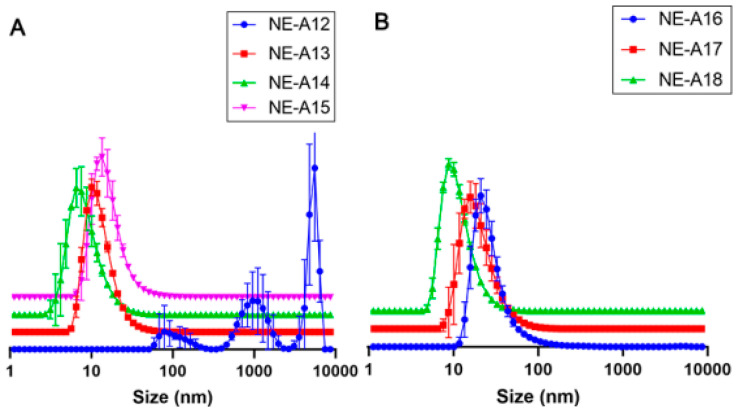
Droplet size distribution curves (nm) for (**A**) NE-A12 to NE-A15 and (**B**) NE-A16 to NE-A18.

The mean sizes, PDIs, and their respective standard deviations for the NEs were developed containing 9% *w*/*w* Tween^®^ 80 and 3% Span^®^ 20, including the NE that presented the best results with ultrasound, that is, NE-A2, while varying the rotation power to 6000 rpm (NE-A16), 10,000 rpm (NE-A17), and 14,000 rpm (NE-A18). The processing of the NEs using the Ultra-Turrax^®^ equipment at 6000 rpm (NE-16) results in a significantly larger droplet size (*p* > 0.05) than that of the systems prepared using ultrasound. The higher the power of this equipment, the smaller the droplet size obtained, as well as its dispersion, which is more homogeneous. Therefore, it is possible to observe that even with high PDIs, NE-A16 presents a mean droplet size of approximately 81 nm, which is more suitable and safer for topical application, and a more uniform distribution curve among the evaluated NEs. The droplet size distribution curves obtained for the NEs are in Figure 2A (NE-A12 to NE-A15) and Figure 2B (NE-A16 to NE-A18).

**Figure 3:**
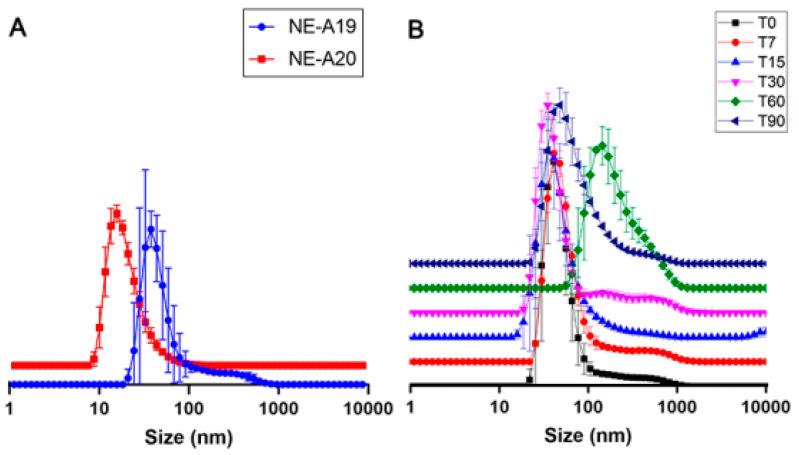
(**A**) Droplet size distribution curves (nm) for NE-A19 and NE-A20. (**B**) Droplet size distribution curves (nm) for NE-A19 during the 90-day stability study.

NE-A19 and NE-A20 nanoemulsions (Figure 3A) were produced based on the results in Figure 2 at a slightly higher concentration of BO in the oil phase (25.31%) to investigate a possible positive impact on droplet sizes and PDIs. In fact, it can be observed that NE-A19 presents a lower dispersion than that of NE-16 (*p* > 0.05), and a larger average droplet size when compared with the other formulations (*p* > 0.05), which represents a lower risk of permeation through the skin according to scientific literature [27,28]; therefore, it was considered the most suitable preparation for proceeding with the long-term stability study (Figure 3B).

In the long-term stability study of NE-A19, desirable mean droplet sizes and PDIs were observed at room temperature (25 ± 2 °C), and were sheltered from sunlight and artificial light. Three samples of 70 mL each were prepared, using the composition of NE-A19, and following the same processing parameters in the Ultra-Turrax^®^ equipment, with the average of this triplicate being obtained for the evaluated times of 0, 7, 15, 30, 60, and 90 days (Figure 3B).

NE-A19 exhibited a non-significant variation in the average droplet size up to 30 days, while the average PDIs were around 0.3 throughout the study, indicating that the sample is good quality. The 60 day analysis showed a significant variation (*p* > 0.05) of these parameters; however, after 90 days, the sample produced results similar to the initial ones (*p* < 0.05), demonstrating the physical stability of NE-A19 for up to 3 months.

In the 60 day study (T60), a signal was visualized in the average droplet size of approximately 210 nm, indicating a possible destabilization of the nanosystem, which was accompanied by a reduction in PDI (by approximately 0.180). However, at the end of the 90 day study (T90), NE-A19 exhibited a compatible average droplet size of approximately 120–160 nm associated with a PDI of approximately 0.300. This range of variation visualized during the first 30 days of analysis (T30) indicates that the results obtained in T60 are not representative of a destabilization of the developed nanosystem, since at the end of the study, NE-A19 showed an average droplet size of approximately 134 nm, which was 9% larger than the original size (approximately 123 nm), and a PDI of 0.314. Based on these results, NE-A19 can be expected to be physically stable over long storage periods at room temperature and protected from light.

The result of the evaluation of the viscosity of NE-A19 is quite interesting and has an impact mainly related to the sensory displayed between the two preparations that were developed on the same day. In fact, there is no standardized relationship of absolute viscosity values to obtain an “ideal preparation”. Readings were taken in triplicates for NE-A19 and a “control” nanoemulsion of the same composition but not having the spray-dried active compound (AV), which was named (NE-B). The obtained results verify that the formulation without AV (NE-B) has a lower viscosity (approximately 273.01 × 10^3^ cps) than the NE containing AV (NE-A19), which exhibits a viscosity of 307.33 × 10^3^ cps (*p* < 0.05). This is attributed to the fact that the spray-dried AV is a powder, and the concentration used represented a considerable volume; thus, the AV powder contributes significantly to the increase in the total viscosity of NE-A19, conferring a creamier appearance and a better sensory effect. 

**Figure 4:**
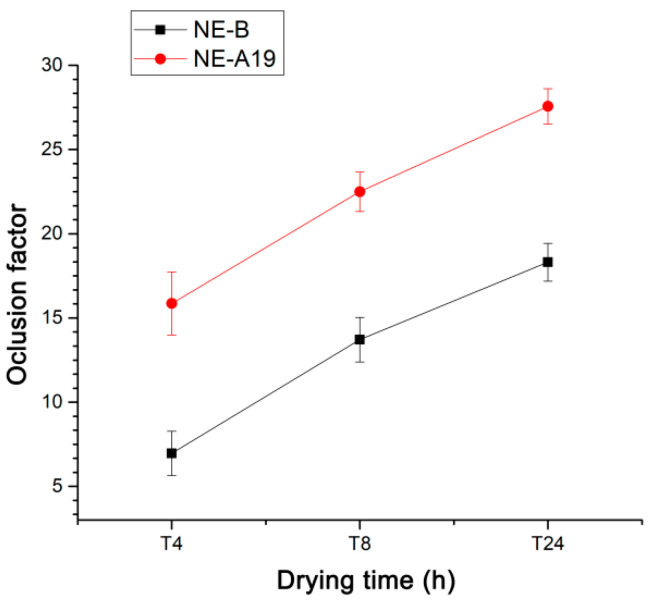
Occlusion factors as a function of the drying time for NE-A19 (red) and NE-B (black) from 4 to 24 h.

Occluded skin can absorb up to five or six times its dry weight in water. Dermocosmetic preparations that demonstrate occlusive capacity tend to delay the loss of transdermal water to the environment due to the formation of a film on the skin. Photoprotective dermocosmetic preparations act as protecting agents against excessive sun exposure [29,30]. The water retention capacity provided by these preparations, associated with sensory characteristics and adhesion to the skin, are important for the consumer. In this way, the occlusive capacity of the selected nanoemulsion (NE-A19) was evaluated and compared with that of NE-B. The incorporation of *Aloe* dry extract contributes significantly to the increase in water retention, which is probably associated with the presence of hygroscopic mono- and poly-saccharide compounds, such as glucomannan. In addition to the contribution of *Aloe*, a large part of the occlusive capacity of the nanostructured system is associated with its base composition. The occlusive effect may have been synergistic due to the BO, as vegetable oils have traditionally been associated with the formation of occlusive films on the skin. The loss of aqueous content from the flasks was verified through the weight variations of the containers, and by calculating their respective occlusion factors, in the intervals of 4, 8, and 24 h of drying in an oven for each preparation, according to the results shown in Figure 4.

The proposed nanostructured system presents a high initial SPF (49 ± 4.97), which is compatible with current commercial preparations. When compared with the control, the developed NE-A19 gives satisfactory results. The nanoemulsion without the *Aloe* dry extract (NE-B) exhibits a mean in vitro SPF, UVA/UVB ratio, and λ_c_ that are lower than those obtained by NE-A19 (*p* > 0.05), which indicates a possible isolated contribution of the extract of AV or a synergistic action of AV with BO and the other synthetic filters present, thereby contributing to the observed increase. However, further studies are needed to confirm this hypothesis.

Over time, the study of photoprotective properties has been promoted to ensure stability in the effectiveness of the most promising nanosystem, NE-A19. Over 90 days, a decrease in the mean value of SPF in vitro was observed, right after the first week (T7) (*p* > 0.05). This probably occurs because soon after the preparation of NE-A19, the nanodroplets begin to settle, thereby generating a natural decrease in SPF and presenting a more homogeneous droplet size profile. However, from this point on, the photoprotective efficacy of NE-A19 remains stable throughout the evaluation period, showing a non-significant reduction (*p* < 0.05). Regarding the UVA/UVB ratio and λ_c_, both factors remain virtually unchanged during the entire period of the NE-A19 stability evaluation. The UVA/UVB ratio remains constant throughout the 90 day study, having been classified as moderate according to the Boot’s Star Rating, where the higher the value, the greater the ability to protect against UVA rays. As for the average values of λ_c_, they are in the range of 350–370 nm; thus, implying that NE-A19 exhibits an intermediate level of protection against UVA radiation. However, as it is a relative value of spectral absorbance, it should not be considered a sensitive measure, such as the SPF or UVA/UVB ratio.

In the safety assessment, the results of in vitro cytotoxicity in NE-A19 keratinocytes at a concentration of 0.32 mg/mL allowed the complete maintenance of cell viability when compared with the negative control (*p* > 0.05). From these data, the cytotoxic concentration necessary to cause 50% cell death (CC50) around 1 mg/mL was calculated. 

**Figure 5:**
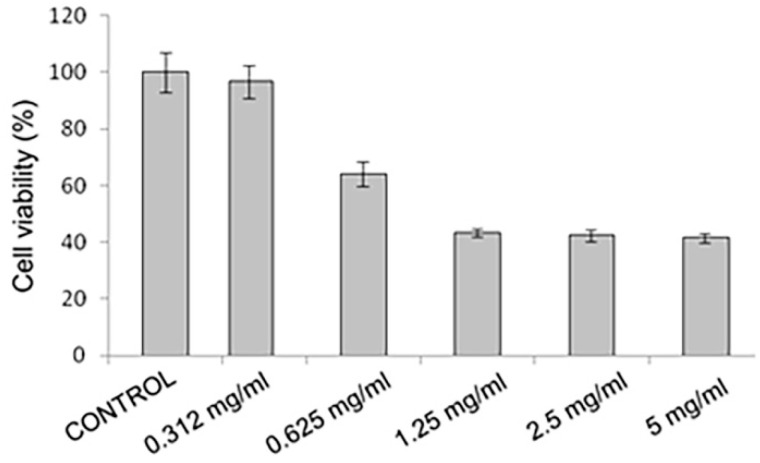
In vitro cytotoxicity analysis using MTT in keratinocytes of the NE-A19 (*p* > 0.05).

These results are very promising for the NE-A19 nanoemulsified system (Figure 5) as it displays a good safety profile for topical application in keratinocytes when compared with several studies in the literature [31,32], which also show CC_50_ values that are in the order of milligrams.

## 3. Materials and Methods

Concentrated dry extract of AV spray-dried 5:1 (Fagron), BO (Plantus, RN, Brasil), octyl-methoxycinnamate (OMC) (Fagron), ethyl-hexyl methoxycrylene (EHMC) (HallStar), sorbitan monolaurate (Span^®^ 20) (Farmos, RJ, Brasil), ethoxylated sorbitan monooleate (Tween^®^ 80) (Farmos), propylene glycol (Galena, Campinas, Brasil), α-tocopherol (Oily Vitamin E) (PharmaNostra, Campinas, Brasil), methylisothiazolinone (Conserve^®^ Novamit) (IPEL, Campo Limpo Paulista, Brasil), and acryloyldimethyltaurate/vinylpyrrolidone (Aristoflex^®^ AVC) (Pharma Special, Itapevi, Brasil).

### 3.1. Equipment and Accessories

UP100H ultrasonic processor, Hielscher (Teltow, Alemanha); Ultra-Turrax^®^ T18-basic Stirrer, IKA (Steufen, Germany); Particle Size Analyzer, Zetasizer Nano ZS, (Malvern, United Kingdom); Digital Scale FA2104N, BIOPRECISA (Curitiba, Brasil); Multifunctional vortex stirrer, model K40-1010, KASVI (São José dos Pinhais, Brasil); UV-2000S Transmittance Analyzer, Labsphere^®^ (North Sutton, USA); Quartz plate (3.5 cm × 3.5 cm); Transpore TM 3M Membrane; Q341-25 distiller, QUIMIS (Diadema, Brasil); Brookfield DV-II Digital Viscometer (Middleboro, USA); Ultrasonic bath, ALTsonic Clean (Piracicaba, Brasil); VERSAMAX Microplate Reader and Molecular Devices (San Jose, USA).

### 3.2. Preparation of o/w Nanoemulsions 

**Figure 6:**
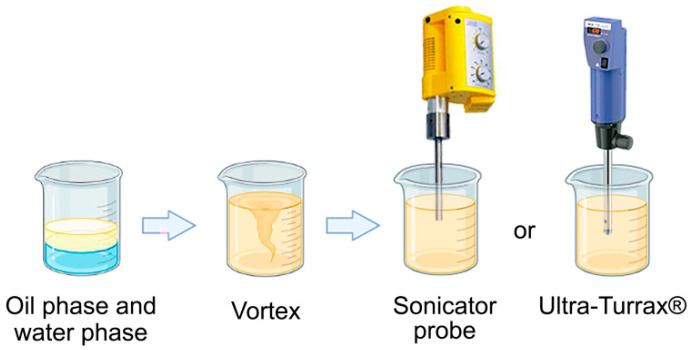
Schematic of o/w NE processing using high-energy methods.

The composition of the proposed nanoemulsions (NEs) presented the usual concentrations of sunscreens in photoprotective preparations already described in the literature [8,33]. Through the use of OMC and EHMC filters to obtain a wide photoprotective spectrum, the concentrations of BO and AV were modulated such that they do not exceed the maximum concentration of a single synthetic filter used, and mainly of the surfactants based on the principle of hydrophilic-lipophilic balance (HLB), which directly influences droplet size and, consequently, the stability and effectiveness of the photoprotective action of nanostructured systems. Two processing methods were evaluated for the preparation of NEs, both of which were high energy, using an ultrasonic processing equipment (US) and an Ultra-Turrax^®^ high-speed homogenizer (Figure 6). The choice of this type of processing was due to the stability characteristics promoted by the high shear caused by these techniques, resulting in more uniform droplet size distributions when compared with those produced using low-energy methods.

The composition of the formulation developed for preparing the NE is shown in Table 1, with the combination of surfactants being varied to determine the most stable nanoemulsion system. 

Synthetic OMC and EHMC chemical filters were used at concentrations of 10% and 3%, respectively, as they are the maximum amounts permitted by current legislation. The 10% BO and AV were added to replace other synthetic filters. The water-soluble surfactants, Tween^®^ 80 and Span^®^ 20, were used in the range of 8–20% to evaluate the stability of the proposed nanostructured system. The use of surfactants was based on the HLB calculation ratio. The 1% Aristoflex AVC^®^ was added to the formulation as a consistency agent. 

### 3.3. Study of Surfactants

The nanoemulsions were prepared via ultrasonication, from NE-A1 to NE-A5, by varying the concentration of Tween^®^ 80 and Span^®^ 20, and for the best proportion of surfactants obtained (Table 2), the variation in the total concentration of surfactants in the formulation (NE-A6 to NE-A8) was evaluated.

### 3.4. Preparation of NEs via Ultrasonic Processing and the Study of Surfactants

In the aqueous phase, the HLB value of Tween^®^ 80 is 15 and that of Span^®^ 20 is 8.6. After evaluating different concentrations of these surfactants, the best proportion for their application in the preparation of NEs was chosen, which corresponded to a HLB value of 7.25 for the BO in the oil phase. The impact of the variation of the ultrasonic processing parameters in the chosen NEs was verified by varying the processing time (min) and amplitude (NE-A9 to NE-A15). The composition of the NEs and the ultrasound parameters used are presented in Table 3.

### 3.5. Preparation of NEs Using Ultra-Turrax^®^

To evaluate the impact of the type of processing, formulations NE-A16 to NE-A20 were prepared. The best proportion of surfactants previously obtained using ultrasound (NE-A6) was used as a basis, while varying the stirring speed of the Ultra-Turrax^®^ and the processing time for each NE. The composition of the nanoemulsions and the parameters of the Ultra-Turrax^®^ used are shown in Table 4.

In NE-A19 and NE-A20, the BO concentration was increased to 12% (*w*/*w*) to verify the impact of a greater amount of oil phase on the particle size. As for the ultrasound-processed NEs, these systems were vortexed for 30 s and then processed in the Ultra-Turrax^®^ for specified periods, with an aliquot being withdrawn every minute within the total processing period of 3 min. 

### 3.6. Determination of Mean Droplet Size and PDI Using Dynamic Light Scattering (DLS) 

The Zetasizer Nano ZS equipment was used for the accomplishment of such determinations, as well as for the evaluation of the particle size and PDI. The NE samples were diluted in distilled water at a 1:100 ratio. The distribution values of mean droplet size smaller than 0.3 and PDI values below 0.7 are indicators of homogeneity and monodispersity in samples [34]. Experiments were performed in triplicates for each sample, with the results expressed as mean ± relative standard deviation. 

### 3.7. Long-Term Stability

The stability of the developed NEs was determined in accordance with the Quality Control Guide for Cosmetic Products of the National Health Surveillance Agency (*Agência Nacional de Vigilância Sanitária*–ANVISA) [35]. The storage conditions of the developed NEs were room temperature (25 ± 2 °C) and protected from light. This study monitored the SPF behavior, considered as the main purpose of the developed NEs, and since they are nanostructured systems, the droplet size and PDI are intrinsically linked to the stability and effectiveness of the NEs [25,33]. The evaluation period was 90 days, with analyses carried out on the 7th (seventh), 14th (fourteenth), 30th (thirtieth), 45th (forty-fifth), 60th (sixtieth), and 90th (ninetieth) days.

### 3.8. Viscosity Determination 

This evaluation consisted of measuring the material resistance to flow using different techniques. The apparent viscosity was read using an eight-speed Brookfield rotary viscometer. The viscometer determined the viscosity encountered by a rotating T-bar needle or spindle immersed in the material. The helipath stand device slowly lowers and raises the rotating needle immersed in the sample, so that the measured resistance is always that of the previously undisturbed substance. As a result, the needle may encounter distinct resistances at different levels, thus recording changes in sample viscosity. Spindle number 4 (E) and speed number 6 were selected. The tests were performed in triplicates at room temperature, with the results expressed as mean ± relative standard deviation.

### 3.9. Evaluation of the Occlusive Potential of NEs

To evaluate the occlusive potential and, consequently, the moisturizing potential of the developed NEs, we applied an analysis method involving the use of 40 mL glass beakers of 4.6 cm in diameter, each filled with 30 g of distilled water, on which a cellulose filter paper membrane (90 mm, Whatman no. 6, cut size of 3 μm) mimicking human skin and fixed using elastic cords, was positioned. The beakers were identified and weighed individually, and the initial reference point of the assay was determined. Thereafter, approximately 200 mg of the sample (13.3 mg/cm^2^) to be evaluated were homogeneously spread on the surface of the membrane to form a film, and the containers were weighed again. Following this, the glasses were stored in a drying oven at 32 ± 2 °C, 50% to 55% relative humidity, for a maximum period of 12 h, and the glasses were weighed in predetermined periods. If the sample evaluated had occlusive properties, a reduction in the loss of aqueous content from the beaker would be observed [36].

Readings were performed in triplicates. The first was a container without any dispersing material on its surface (control). The second was in a container in which the developed NE did not contain the main active component, the concentrated dry AV extract was spread. The third was a container in which the developed NE contained the concentrated dry AV extract. This approach aimed at evaluating the impact on the occlusive potential of both the nanostructured system and the active compound used. Subsequent weighing procedures were performed from time zero (T0) at intervals of 4 and 8 h up to the maximum period of 24 h. The occlusion factor (F) of the sample is calculated using Equation (1):

F = [(A − B)/A] × 100(1)

where A represents the water loss without the sample (blank) and B the water loss with the sample.

### 3.10. Determining the SPF, Critical Wavelength, and UVA/UVB Ratio Using Transmittance Spectrophotometry with Integrated Sphere

In vitro determination of the SPF via spectrophotometry with integrated sphere was carried out using UV-2000S Labsphere^®^ equipment, in which a sample is positioned on a quartz plate, functioning as a substrate that is ideally transparent in the UV range, with texture and porosity similar to human skin. The literature recommends alternative complementary substrates to meet all these requirements. Surgical tape (TransporeTM, 3M), a polyvinylidene chloride film (Saran Wrap^®^), and collagen membrane covering the quartz plate for meeting the experimental conditions. The quartz plate was prepared with approximately 50 mg of glycerin to obtain a base line in the equipment. The next step was the deposition of approximately 50 mg of the evaluated NE sample on the plate, covered by the film, spread with the help of a latex fingertip, aiming at obtaining the most uniform and homogeneous layer possible for the equipment reading. This reading was performed at nine different points on the quartz plate. The plate was placed on a metal support, which is taken to the equipment and receives the UV light. The SPF readings were made in triplicates for calculating the mean ± relative standard deviation. 

The critical wavelength is a spectrophotometrically-determined value based on spectral absorbance and is used to assess whether a photoprotector offers UVA protection. Because it is a relative value–not an absolute value-of spectral absorbance, it is not considered a sensitive measure, such as SPF or that obtained using Boot’s Star Rating (Table 5). For the analysis, the measured spectral transmittance was converted into spectral absorbance, where the ratio (R) was calculated. The critical wavelength is defined as the first value found when the value of R is > 0.9, that is, the wavelength for which the area under the integrated optical density curve, which starts at 290 nm, is equal to 90% of the integrated area between 290 and 400 nm. Therefore, the value of the critical wavelength is related to the level of protection, in which λ_c_ values between 340 and 370 nm indicate intermediate protection against UVA radiation, and values above 370 nm indicate greater protection over a wide spectrum [37].

Boots Star rating measured the % of UVA that’s been absorbed compared to UVB rays (source), so in a sense it measured the evenness of the UV protection. The closer the UVA/UVB ratio is to 1, the more stars a sunscreen gets. Five stars on the Boots system means that UVA protection achieved more than 90% UVB protection.

### 3.11. Evaluation of In Vitro Cytotoxicity Using the [3-(4,5-Dimethylthiazol-2yl)-2,5-diphenyl Tetrazolium Bromide Method (MTT)

In the evaluation of the cytotoxicity of the developed NEs, the HaCaT human keratinocyte lineage (CLS–Cell Line Service GmbH, Eppelheim, Germany) was used. These cells were cultivated in a DMEM medium (Dulbecco’s Modified Eagle Medium) with 10 g/L of supplemented glucose, 100 UI/mL of penicillin, and 100 µg/mL of streptomycin. The cells were maintained in 75 cm^2^ culture bottles in an oven at 37 °C and under an atmosphere of 5% CO_2_. When the cultures reached confluence, the cells were washed with PBS (Phosphate Buffered Saline) (2.67 mM KCl, 137.93 mM NaCl, 1.47 mM KH_2_PO_4_, and 8.10 mM Na_2_HPO_4_; pH 7.2) and incubated in 0.25% trypsin and 0.2 g/L EDTA solution for about 10 min at 37 °C to resuspend them in solution. After trypsinization, DMEM with 10 g/L of glucose supplemented with 10% FBS (Fetal Bovine Serum) was used to inhibit the action of trypsin. The cells were then harvested via centrifugation at 2000 rpm for 5 min at room temperature. The pellet formed by the cells was resuspended in DMEM with 10 g/L of glucose supplemented with 10% FBS and the cells were counted in a Neubauer chamber. The cells were then plated in a 96-well plate at a concentration of 10^4^ cells per well inside a flow chamber and with preservatives in the sample, requiring no sterilization, so as not to break the nanodroplets. After 24 h, the cells were incubated with different concentrations of the NE sample (5 mg/mL, 2.5 mg/mL, 1.25 mg/mL, 0.625 mg/mL, and 0.312 mg/mL) and diluted in the culture medium for another 24 h in an oven at 37 °C with 5% CO_2_.

Cytotoxicity was evaluated using the MTT method. The medium was discarded and 100 µL of the MTT reagent (reagent prepared in a PBS w/EDTA solution at a final concentration of 0.5 mg/mL) was added in the dark. The plate was incubated by being wrapped in aluminum foil for approximately 2 h 30 min under the same incubation conditions described above. The absorbance reading was performed in triplicates in a spectrophotometer (ELISA) at 570 nm [38].

### 3.12. Statistical Analysis

The experimental results obtained were expressed as mean ± standard deviation and were submitted for statistical analysis using Origin^®^ software version 8.0 for Windows. Also, the statistical analysis of variance (ANOVA) was performed with statistical significance at the level of 95% (α = 0.05).

## 4. Conclusions

Currently, the search for eco-friendly products from renewable sources which have been not tested on animals and are prepared using innovative technological processes has become the target of the cosmetic industry and the main interest of the consumer market.

The developed nanoemulsions, with BO and spray-dried AV, presented pleasant appearance and sensory characteristics. The variation in the concentration of surfactants and their total amount drastically influenced the average droplet size, PDI, and physical stability of the developed NEs. It was demonstrated that processing using the Ultra-Turrax^®^ was most efficient in the preparation of the proposed nanostructured system, at 6000 rpm for 3 min to produce NE-A19, the most promising nanoemulsion with an average droplet size of 146.80 ± 2.74 nm and monomodal dispersion. Furthermore, regarding the size distribution and PDI, NE-A19 exhibited a high degree of long-term stability (T = 90 days) at room temperature. The sensorial characteristics were demonstrated in the evaluation of the viscosity of NE-A19, being superior to that of NE-B of the same composition, but in the absence of the main active compound, the spray-dried *Aloe vera* extract, conferring adequate sensorial characteristics to the nanostructured system, which is a very important factor for consumer acceptance. Regarding occlusive potential, NE-A19 presented 27.56 ± 1.04, a result that is above that of NE-B, which was 18.30 ± 1.12 after 24 h of drying (*p* < 0.05). In this case, it was inferred that the presence of *Aloe* dry extract contributed significantly to this increase.

The SPF ± relative standard deviation of NE-A19 nanoemulsions was 49.00 ± 4.97, and for NE-B it was 42.0 ± 2.16. These were considered satisfactory when compared with traditional formulations, indicating the viability of nanoemulsion systems for photoprotective activity. Regarding the SPF, during the entire period of stability of the nanoemulsified systems, at room temperature, there was a slight decrease over the 90 days, in addition to a stable UVA/UVB ratio and critical wavelength in an indicative range of moderate protection against UVA radiation.

Additionally, NE-A19 exhibited a good safety profile against the cells in the epidermis— the keratinocytes—presenting a CC_50_ that was in the order of milligrams and, therefore, can be considered safe for topical use. Therefore, NE-A19 proved to be promising as a vehicle for sunscreens. In addition, BO and spray-dried AV extract exhibited a synergistic effect on the stability and cytotoxicity of NE-A19, demonstrating safety for topical application, and requiring further studies to evaluate its efficacy profile relative to that of traditional formulations.

## Figures and Tables

**Table 1:** General composition of the developed NEs.

	Raw Material	Concentration (%)
Oil Phase	Buriti oil	10
	Octyl Methoxycinnamate (OMC)	10
	Ethylhexyl methoxycrylene (EMC)	3
	Conserve^®^ Novamit	3
	α-tocopherol	0.01
	*Aloe vera spray dryed (AV)*	10
Water Phase	Aristoflex AVC^®^	1
	Propyleneglycol (PROP)	2
	Tween^®^ 80	8–20
	Span^®^ 20	
	Distilled water	q.s.

**Table 2:** Composition of the prepared NEs and variations in surfactant concentration (NE-A1 to NE-A8).

NE	Concentration				Parameters	
	Tween^®^ 80	Span^®^ 20	Oil Phase	Water Phase	Time (min)	Amplitude (%)
Surfactant Study						
NE-A1	12	3	23.31	q.s. 100%	25	100
NE-A2	9	6	23.31		25	100
NE-A3	7.5	7.5	23.31		25	100
NE-A4	6	9	23.31		25	100
NE-A5	3	12	23.31		25	100
NE-A6	6	4	23.31		25	100
NE-A7	12	8	23.31		25	100
NE-A8	4.8	3.2	23.31		25	100

**Table 3:** Composition of NEs and ultrasound parameters (NE-A9 to NE-A15).

NE	Concentration (%)				Parameters	
	Tween^®^ 80	Span^®^ 20	Oil Phase	Water Phase	Time (min)	Amplitude (%)
Surfactant Study						
NE-A9	9	6	23.31	q.s. 100%	10	100
NE-A10	9	6	23.31		15	100
NE-A11	9	6	23.31		20	100
NE-A12	9	6	23.31		0	20
NE-A13	9	6	23.31		1	20
NE-A14	9	6	23.31		2	20
NE-A15	9	6	23.31		3	20

**Table 4:** Composition of NEs, variation of the BO concentration (NE-A19 and NE-A20) and Ultra-Turrax^®^ speed (NE-A16 to NE-A20).

NE	Concentration (%)				Parameters	
	Tween^®^ 80	Span^®^ 20	Oil Phase	Water Phase	Time (min)	Velocity (rpm)
NE-A16	9	6	23.31	q.s. 100%	3	6000
NE-A17	9	6	23.31		3	10,000
NE-A18	9	6	23.31		3	14,000
NE-A19	9	6	23.31		3	6000
NE-A20	9	6	23.31		3	10,000

**Table 5:** Boot’s Star rating system.

Anti UVA Protection		
UVA/UVB Ratio	Stars	Description
0.0 to < 0.2	-	Too low
0.2 to < 0.4	*	Moderate
0.4 to < 0.6	**	Good
0.6 to < 0.8	***	Superior
0.8 to < 0.9	****	Maximum
≥ 0.9	*****	Ultra

## Data Availability

Data is contained within the article.

## Supplementary Materials

The following supporting information can be downloaded at: https://www.mdpi.com/article/10.3390/ph16020292/s1, Figure S1: NE-A19 nanoemulsion development flowchart.
