# Supplementary figures and images for: Nanotechnology as a Tool for Optimizing Topical Photoprotective Formulations Containing Buriti Oil (Mauritia flexuosa) and Dry Aloe vera Extracts: Stability and Cytotoxicity Evaluations
Source: Pharmaceuticals (Basel). 2023 Feb 14;16(2):292. doi: 10.3390/ph16020292 (PMC9968176; doi:10.3390/ph16020292)

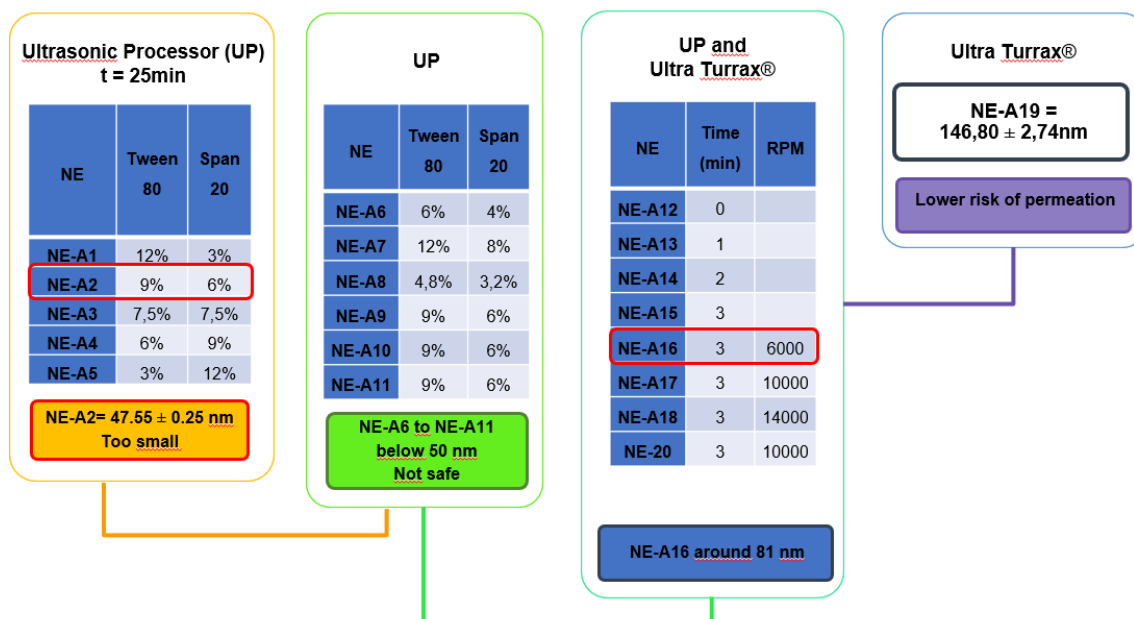

Figure S1. NE-A19 nanoemulsion development flowchart.

Supplement: Supplementary file 1 [file pharmaceuticals-16-00292-s001.zip › pharmaceuticals-2161957-supplementary.pdf]
